# Supplementary figures and images for: Macrophage Phenotype Induced by Circulating Small Extracellular Vesicles from Women with Endometriosis
Source: Biomolecules. 2024 Jun 21;14(7):737. doi: 10.3390/biom14070737 (PMC11274790; doi:10.3390/biom14070737)

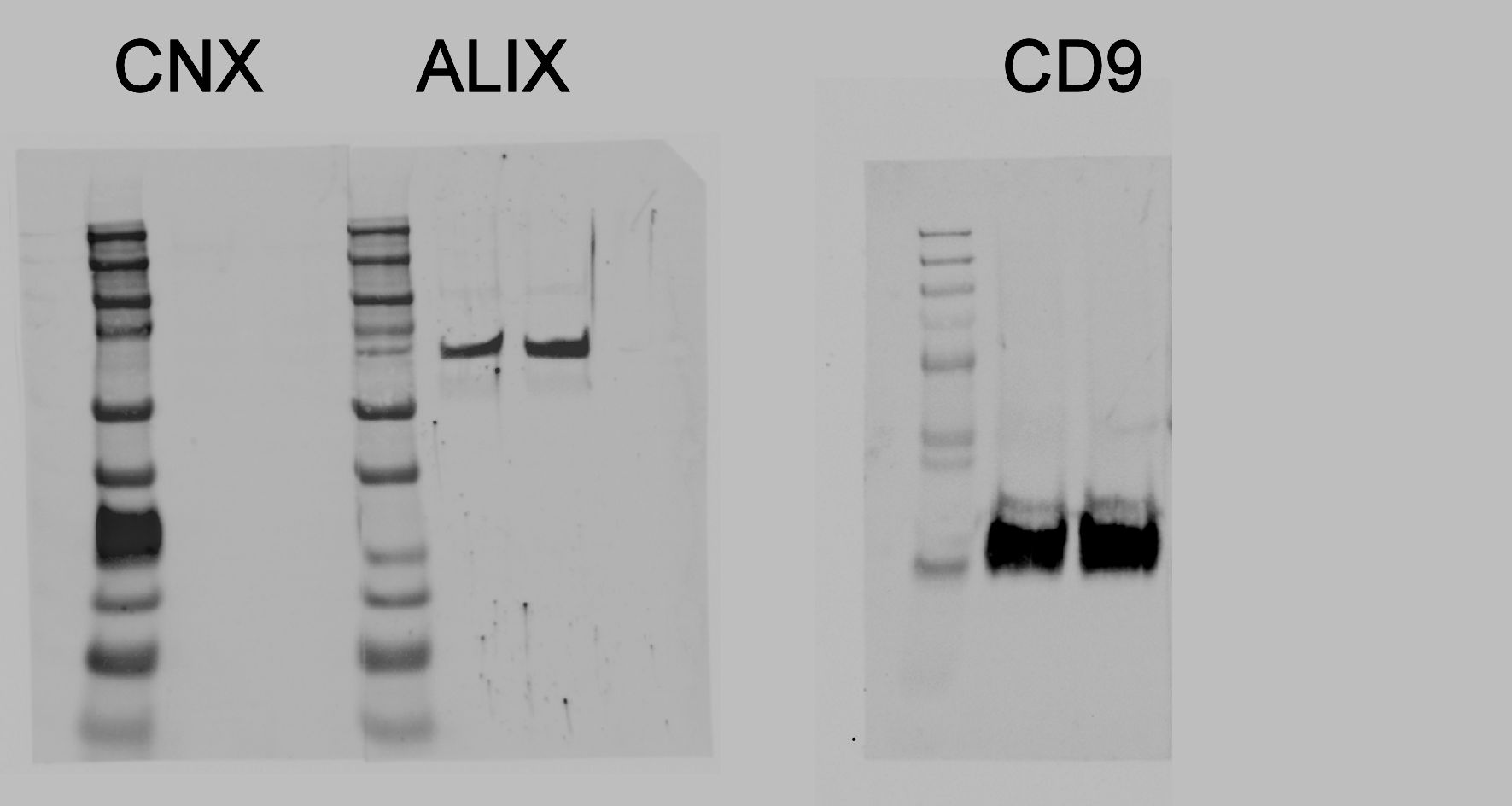

Supplement: Supplementary file 1 [file biomolecules-14-00737-s001.zip › biomolecules-3007498-supplementary.jpg]
